# Coronary Reaccess After Modified Chimney Stenting in a Self-Expanding Transcatheter Valve-in-Valve

**DOI:** 10.1016/j.jaccas.2023.102118

**Published:** 2023-11-15

**Authors:** Won-Keun Kim, Chris Frawley, Efstratios I. Charitos, Arif A. Khokhar

**Affiliations:** a Department of Cardiology, Kerckhoff Heart Center, Bad Nauheim, Germany, and German Center for Cardiovascular Research, Partner Site Rhine-Main, Frankfurt am Main, Germany; b Department of Cardiac Surgery, Kerckhoff Heart Center, Bad Nauheim, Germany; c Department of Cardiology, Justus-Liebig University of Giessen, Giessen, Germany; d Boston Scientific, Galway, Ireland; e Department of Cardiology, Hammersmith Hospital, Imperial College Healthcare NHS Trust, London, United Kingdom

**Keywords:** ACURATE valve system, chimney stenting, coronary re-access, valve-in-valve

## Abstract

We demonstrate a modified technique of heterotopic chimney stenting for coronary obstruction during valve-in-valve transcatheter aortic valve replacement With successful end-on cannulation via the stent ostium. Our technique was reproducible on the bench with successful reaccess and without any interaction between the deployed coronary stent and the prosthetic leaflets.

## Graphical abstract

**Figure:**
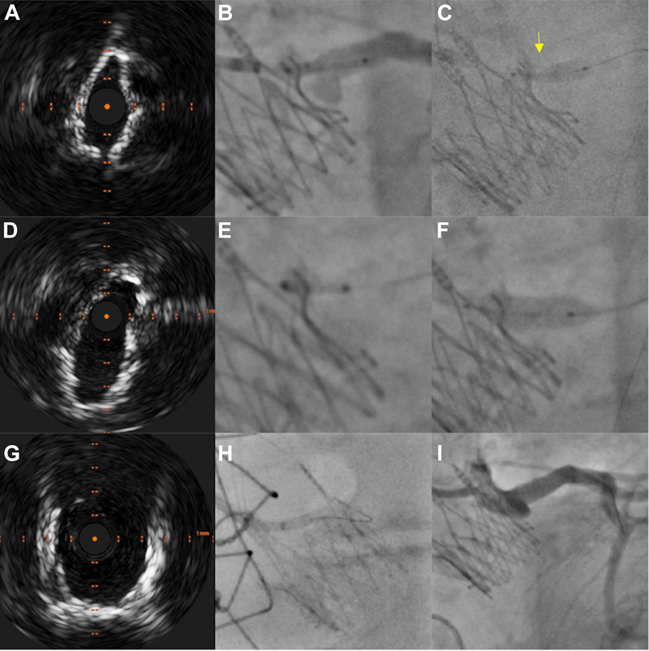

A 77-year-old woman was admitted with decompensated heart failure caused by failure of a Trifecta 23-mm surgical aortic valve from 2008 with severe stenosis (mean gradient 38 mm Hg, effective orifice area 0.8 cm^2^). Despite intravenous diuretic agents, the patient’s condition deteriorated into incipient cardiogenic shock with increasing need for vasoactive pressure support. Therefore, a decision was made for emergent transcatheter aortic valve replacement (TAVR). Preprocedural computed tomography confirmed an average diameter of 21 mm of the surgical ring. The distances from the basal ring to the left coronary artery (LCA) and right coronary artery (RCA) were 3.7 mm and 8.6 mm, respectively. The virtual valve-to-coronary distance to the LCA and RCA were 2.8 mm and 1.7 mm, respectively.

**Learning Objectives**

• To recognize that with this modified technique of snorkel stenting during valve-in-valve TAVR, coronary access via the stent ostium becomes feasible.
• To adopt the technique with all components that contribute to coronary re-access, including commissural alignment, implantation of a second stent, and flaring of the stent ostium.
• To motivate physicians to applying the modified snorkel stenting technique during valve-in-valve TAVR.

The procedure was performed under mild analgesic sedation via transfemoral access. For coronary protection of the left main, we positioned a 4.0/15-mm drug-eluting stent in the LCA via a 6-F Judkins left 3.5 catheter. After successful implantation of an ACURATE neo2 S valve (Boston Scientific) using commissural alignment, selective angiography of the LCA showed a sufficient distal flow, but intravascular ultrasound (IVUS) revealed a relevant obstruction at the ostial left main with a minimal luminal area of 3.8 mm^2^ (Figure 1A). Therefore, the drug-eluting stent was deployed in modified chimney technique by placing the ostium of the stent within the upper crown (Figures 1B and 1C). Despite proximal optimization using a 5.0/15 noncompliant balloon, a slit-shaped narrowing at the proximal stent part remained (Figure 1D). Hence, a second drug-eluting stent was implanted (Figure 1E), followed by aggressive postdilation to flare the ostium using a 6.0/20 mm noncompliant balloon (Figure 1F). During withdrawal of the bulky balloon catheter, the coronary wire was removed inadvertently from the coronary artery. The guiding catheter had to be re-engaged and was placed in front of the stent ostium with the support of the guide extension. After several attempts, it was possible to rewire the coronary stent via the true ostium (Figure 1H, Video 1). Final angiography showed a good angiographic result with TIMI flow grade III (Figure 1I). The correct orthotopic position with end-on cannulation of the coronary wire was verified on IVUS. The minimal luminal area of the stent in the compressed section exceeded 10.0 mm^2^, whereas the ostial stent portion was widely flared (Figure 1G).

**Figure 1:**
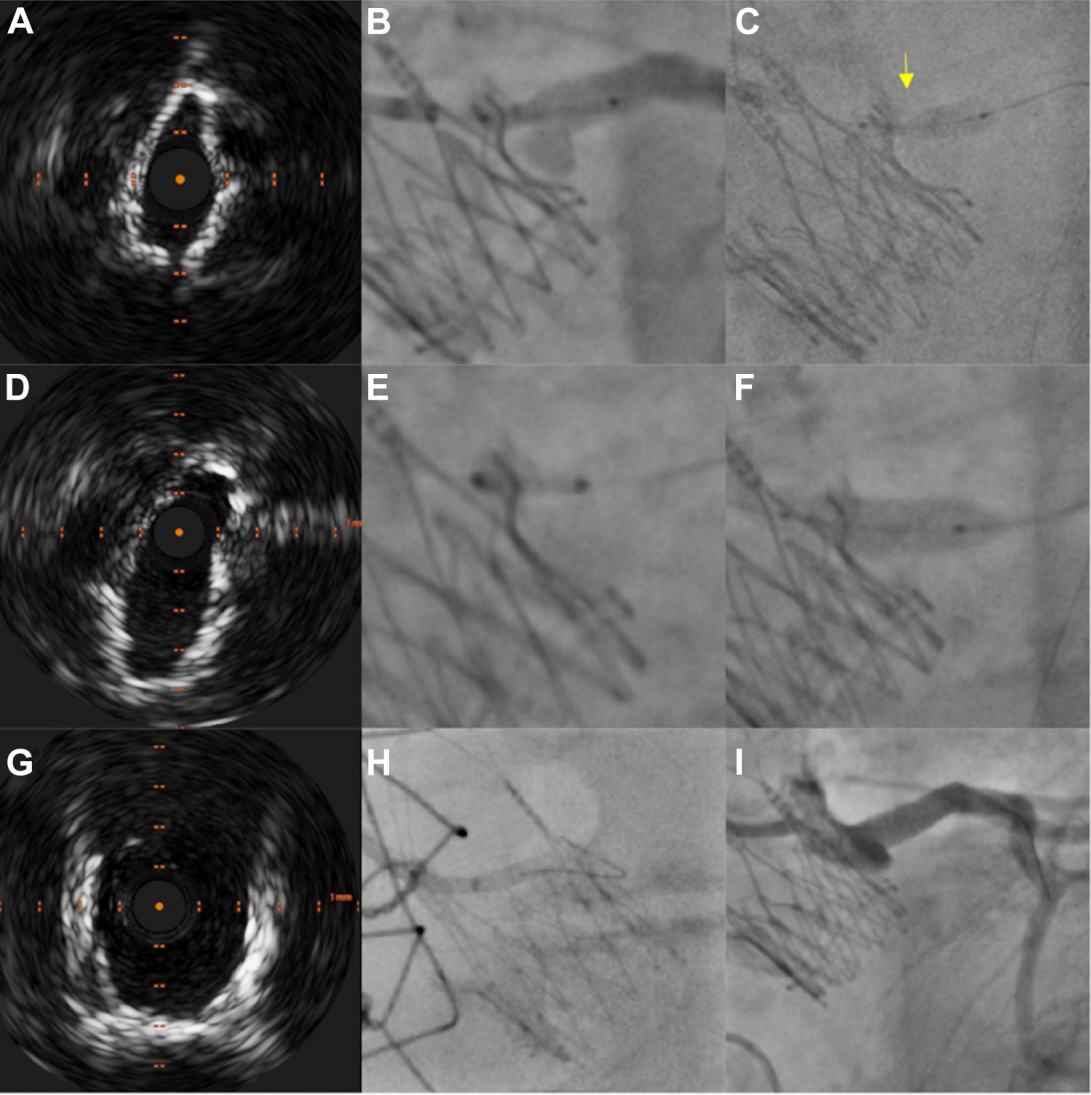
Chimney Stenting and Intravascular Ultrasound Relevant left main ostial obstruction on intravascular ultrasound (IVUS) (A). Positioning of the first coronary stent within the upper crown of the ACURATE neo2 prosthesis (B). Distinct narrowing during inflation of the coronary stent (yellow arrow, C). Owing to a slit-like narrowing of the first deployed coronary stent (D), a second coronary stent was implanted (E). After proximal optimization (F) there was a good final result with widely flared ostial stent portion (G). After inadvertent withdrawal of the guiding catheter, successful selective re-engagement and insertion of a coronary wire for final IVUS assessment (H) with patent left coronary artery and TIMI flow grade III (I).

The patient recovered gradually and could be weaned from vasopressor medication. On echocardiography before her discharge, there was no aortic regurgitation, and the mean gradient was 11 mm Hg. Post-TAVR computed tomography showed commissural alignment of the prosthesis and an optimal position of the coronary stent ostium that was embedded in the upper crown without any obvious contact with the prosthetic leaflets (Figures 2A and 2B). The patient was discharged in stable condition 2 weeks after the procedure.

**Figure 2:**
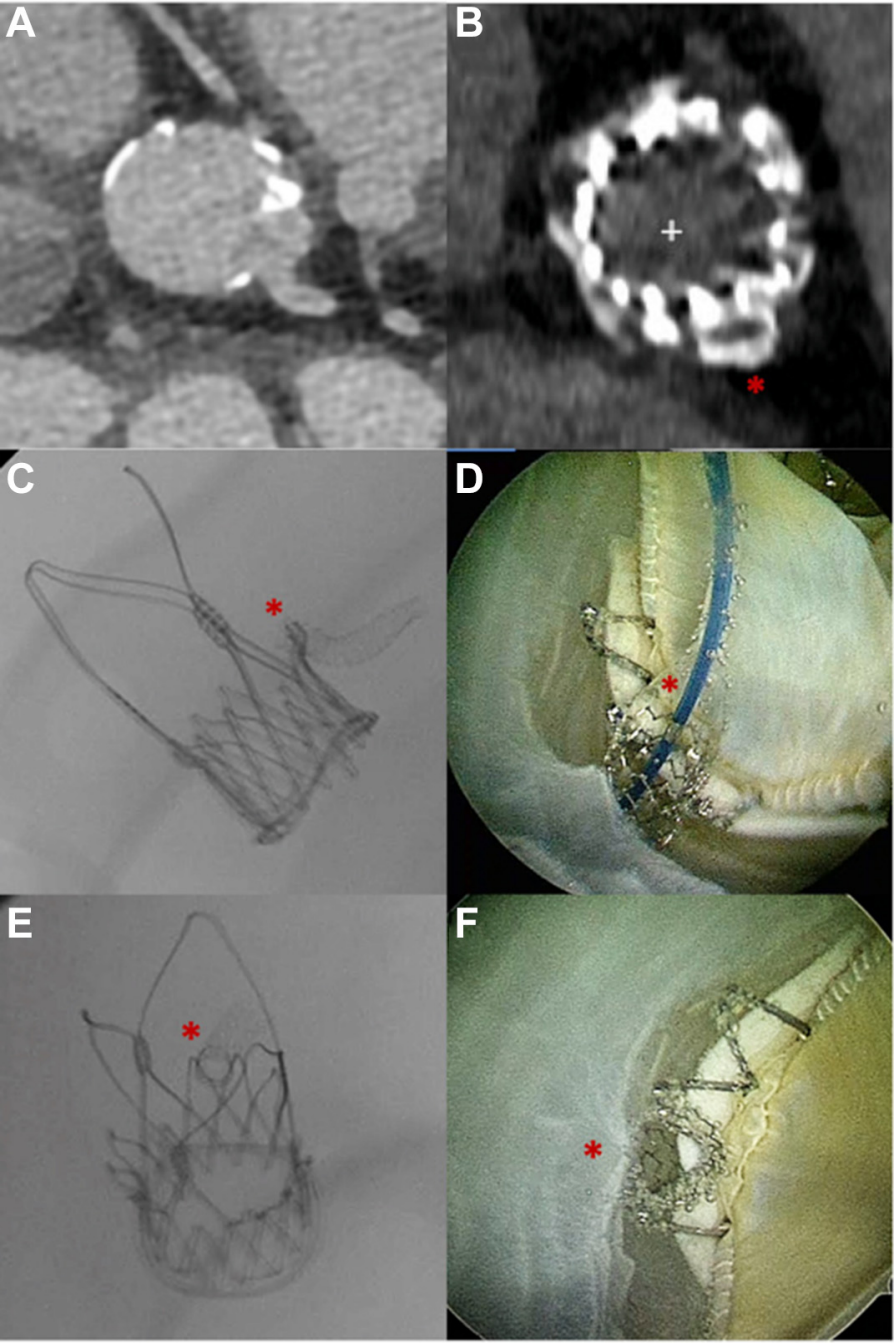
Pre- and Post-TAVR Computed Tomography and Bench Testing Computed tomography (CT) before transcatheter aortic valve replacement (TAVRU) showing left main without stenosis (A). Post-TAVR CT showing the ostium of the coronary stent (red asterisks) embedded in the upper crown (B). Patient-specific bench test simulation with chimney stenting (C) and successful end-on cannulation of the coronary stent (D). The ostial portion of the stent is widely flared both on fluoroscopy (E) and on the borescope camera (F).

## Bench test results

A patient-specific 3-dimensional print model of the aortic root was used to simulate the implantation of an ACURATE neo2 size S in a Trifecta 23-mm surgical aortic valve with subsequent chimney stenting using the modified technique.^1^ Coronary reaccess was reproducible with end-on cannulation of the coronary stent, as shown on high-resolution borescope camera images (Figures 1C to 1F, Video 2). Notably, there was no relevant interaction between the deployed coronary stent and the prosthetic leaflets (Video 3).

## Discussion

We demonstrate a modified technique of chimney stenting for coronary obstruction during valve-in-valve TAVR that could facilitate coronary re-access via the stent ostium. To the best of our knowledge, this is the first reported case of a successful end-on cannulation after chimney stenting. Giannini et al^2^ reported unsuccessful attempts at orthotopic coronary re-access after standard chimney stenting in a bench model with an ACURATE and EVOLUT valve inside a Perimount prosthesis. Instead, only side-on cannulation was possible, which would require crushing of the stent to enable crossing with a new stent. Our technique could make chimney stenting more acceptable among clinicians because it is less complex than the BASILICA procedure. The factors that may facilitate coronary re-access include commissural alignment, embedding of the coronary stent ostium within the upper crown along with profound flaring using a large balloon, and implantation of a second stent. The second stent adds significantly to the longitudinal strength and prevents compression during re-engagement, and the double layer will help prevent unwanted side access into the stent. Hence, it may be more appropriate to refer to this technique as heterotopic snorkel stenting. The feasibility of coronary re-access using our modified technique was confirmed in a bench model with patient-specific simulation.

## Conclusions

We present a modified technique of heterotopic snorkel stenting: 1) that facilitates future coronary reaccess; 2) without interaction between the heterotopic stent and valve leaflets; and 3) minimizes interaction between the heterotopic stent and valve frame, which reduces the chances of stent distortion and subsequent late stent failure.

## Funding Support and Author Disclosures

Dr Kim has received personal fees from Abbott, Boston Scientific, Edwards Lifesciences, Meril Life Sciences, and Shockwave; and has received institutional fees from Boston Scientific. Mr Frawley is an employee of Boston Scientific. Dr Charitos has been a proctor for Boston Scientific. Dr Khokhar has received speaker fees from Boston Scientific.

## Appendix

Video 1 Successful Re-Engagement and Insertion of Coronary Wire Into Chimney Stent Via End-on Cannulation

Video 2 Successful End-on Cannulation of Chimney Stent Documented by High-Resolution Borescope Camera in Bench Test

Video 3 High-Resolution Borescope Camera Video Showing No Interaction Between Deployed Coronary Stent and Prosthetic Leaflets
